# Sustainable deep vision systems for date fruit quality assessment using attention-enhanced deep learning models

**DOI:** 10.3389/fpls.2025.1521508

**Published:** 2025-06-30

**Authors:** Esraa Hassan, Sarah Abu Ghazalah, Nora El-Rashidy, Tarek Abd El-Hafeez, Mahmoud Y. Shams

**Affiliations:** ^1^ Faculty of Artificial Intelligence, Kafrelsheikh University, Kafrelsheikh, Egypt; ^2^ College of Computer Science, King Khalid University, Abha, Saudi Arabia; ^3^ Computer Science Unit, Deraya University, Minia, Egypt; ^4^ Department of Computer Science, Faculty of Science, Minia University, Minia, Egypt

**Keywords:** fruit classification, DenseNet121, Squeeze-and-Excitation, YOLOv8n, augmentation, segmentation

## Abstract

**Introduction:**

Accurate and automated fruit classification plays a vital role in modern agriculture but remains challenging due to the wide variability in fruit appearances.

**Methods:**

In this study, we propose a novel approach to image classification by integrating a DenseNet121 model pre-trained on ImageNet with a Squeeze-and-Excitation (SE) Attention block to enhance feature representation. The model leverages data augmentation to improve generalization and avoid overfitting. The enhancement includes attention mechanisms and Nadam optimization, specifically tailored for the classification of date fruit images. Unlike traditional DenseNet variants, proposed model incorporates SE attention layers to focus on critical image features, significantly improving performance. Multiple deep learning models, including DenseNet121+SE and YOLOv8n, were evaluated for date fruit classification under varying conditions.

**Results:**

The proposed approach demonstrated outstanding performance, achieving 98.25% accuracy, 98.02% precision, 97.02% recall, and a 97.49% F1-score with DenseNet121+SE. In comparison, YOLOv8n achieved 96.04% accuracy, 99.76% precision, 99.7% recall, and a 99.73% F1- score.

**Discussion:**

These results underscore the effectiveness of the proposed method compared to widely used architecture, providing a robust and practical solution for automating fruit classification and quality control in the food industry.

## 1 Introduction

Agricultural automation has become a cornerstone in addressing the growing demand for efficient and sustainable farming practices (Vinod et al., 2024). Accurate and automated fruit classification is critical for enhancing productivity, ensuring quality control, and optimizing processes such as yield prediction, disease detection, and crop management (Sharma and Shivandu, 2024). Among various crops, date fruits hold significant economic and cultural value, particularly in arid regions (Al-Mssallem et al., 2024). However, the variability in fruit appearance, including differences in size, shape, color, and maturity stage, presents considerable challenges for accurate classification (Hameed et al., 2018; Hassan, 2024). These challenges are further compounded by the complexities of capturing images in real-world farm environments, where dynamic conditions such as varying lighting, diverse backgrounds, and occlusions can hinder the reliability and robustness of classification models (Tang et al., 2023).

Despite advancements in computer vision and deep learning (DL), existing solutions often struggle to achieve high accuracy and generalizability under such unconstrained conditions (Ojo and Zahid, 2022). While models like VGG, GoogleNet, MobileNet, and EfficientNet have shown promise, their performance is often limited when applied to multi-class classification of fruit varieties (Yu et al., 2023). Furthermore, the classification of multiple date fruit cultivars, each exhibiting subtle yet distinct visual characteristics, underscores the need for a specialized model capable of handling these intricate differences (Khalil et al., 2017).

### 1.1 Research motivation and gaps

This paper is motivated by the pressing need for an effective and practical solution to automate the classification of date fruits across diverse varieties and conditions (Hassan et al., 2021; Abouelmagd et al., 2024; Elmessery et al., 2024). Current approaches face significant gaps, including:

- The lack of robust models that can effectively handle the inherent variability in fruit appearance under real-world farm environments (Zhang and Yang, 2024).
- Insufficient utilization of attention mechanisms to focus on critical image features, which limits the ability to distinguish between similar cultivars (Niu et al., 2021).
- The absence of models optimized for multi-class classification of date fruit varieties while maintaining generalization across varying conditions (Sultana et al., 2024).

### 1.2 Research question

How can a deep learning-based framework be designed to accurately and reliably classify multiple date fruit varieties under unconstrained real-world conditions, overcoming challenges such as variability in appearance, lighting, and occlusions?

### 1.3 Proposed approach and contributions

To address these challenges, we propose a novel approach to image classification by integrating a DenseNet121 model pre-trained on ImageNet with a Squeeze-and-Excitation (SE) Attention block. This integration enhances feature representation by enabling the model to focus on the most critical aspects of the input images. The use of data augmentation ensures improved generalization and reduces overfitting, making the model suitable for real-world applications. Additionally, the utilization of YOLOv8n algorithm is employed to further refine the model’s learning process, achieving exceptional performance in classifying date fruit images. The key contributions of this paper are as follows:

- Integration of SE Attention Mechanism – This study enhances DenseNet121 with a Squeeze-and-Excitation (SE) attention block, improving feature representation and enabling the model to focus on critical image features.
- Optimization for Fruit Classification – The model is specifically tailored for date fruit classification, leveraging Nadam optimization to improve training efficiency and convergence.
- Enhanced Generalization through Data Augmentation – Various data augmentation techniques were employed to improve model generalization and reduce overfitting, ensuring robustness under diverse conditions.
- Comprehensive Performance Evaluation – Multiple deep learning models, including DenseNet121+SE and YOLOv8n, were compared, demonstrating superior classification accuracy with DenseNet121+SE (98.25%) and YOLOv8n (96.04%).
- High-Accuracy Automated Classification – The proposed approach achieved state-of-the-art performance in precision, recall, and F1-score, making it highly suitable for automated fruit classification and quality control applications in the food industry.
- Potential for Real-Time Implementation – The efficiency of YOLOv8n highlights its applicability for real-time classification, paving the way for future deployment in smart agriculture and food processing systems.

The rest of this paper is organized as follows: Section 2 reviews related works, while Section 3 covers the preliminaries. Section 4 presents the proposed approach, followed by experimental results and analysis in Section 5. The Finally, Section 6 concludes the study.

## 2 Related works

Deep learning techniques, particularly YOLO variants and lightweight CNNs, are widely used in fruit detection (Zhong et al., 2024; Li et al., 2024). While they offer high accuracy and interpretability, they face limitations like limited datasets and specific fruit types (Gulzar et al., 2024). **Table 1** provides a comprehensive overview of the advancements, methodologies, and challenges in fruit image recognition, offering valuable insights for researchers and practitioners in precision agriculture. Sultana et al. (2024) suggest a XAI-FruitNet that is a hybrid deep learning architecture that enhances feature discrimination and achieves over 97% accuracy in fruit classification across various datasets. Its built-in interpretability enhances transparency and trust, setting a new standard for explainable artificial intelligence in agricultural applications. This robust and trustworthy solution is essential for modern agricultural needs. Marin et al. (Marin and Radoi, 2022) present a Convolutional Neural Networks solution for classifying fruits as healthy or damaged, using YOLOv3, a state-of-the-art network for object detection in images. The algorithm achieved over 75% classification accuracy, and the dataset is made available. Huang et al. (2025) propose a fruit recognition and evaluation method using multi-model collaboration. The detection model locates and crops fruit areas, while the cropped image is input into a classification module. The feature matching network optimizes classification results. The improved YOLOv8 model improves P, R, mAP50, and MAP50–95 indicators. The Swin Transformer model has 92.6% accuracy on 27 fruit categories. The study by (Wu et al., 2024) presented an enhanced CycleGAN with a generator using ResNeXtBlocks and optimized upsampling, aimed at improving nighttime pineapple detection. It reduces the Fréchet Inception Distance (FID) score by 29.7% and, when combined with YOLOv7, improves precision by 13.34%, recall by 45.11%, average precision by 56.52%, and F1 score by 30.52%, enabling more accurate detection in low-light conditions. Rao Jerripothula et al. (2021) examine fruit image classification from agricultural, market, and automation perspectives, using pre-trained neural networks and machine learning algorithms. The study achieves 96%, 94%, and 86% accuracies on the novel dataset named RipeRaw, based on bias/variance analysis. Chiagoziem et al. (Ukwuoma et al., 2022) discuss various deep learning methods used for fruit detection and classification, including datasets, practical descriptors, model implementation, and challenges. It also reviews recent studies and a new model for fruit classification using the popular dataset “Fruit 360” for beginner researchers. Zarouit et al. (2024) provides a comprehensive collection of images and videos for detecting, classifying, analyzing, and harvesting dates at various maturity stages, thereby enhancing agriculture research by automating pre and post-harvesting tasks. Almutairi et al. (2024) use Deep Learning to detect and classify date fruits using the You Only Look Once (YOLO) algorithm. YOLOv8 achieved a mean recall of 99.00% and precision of 99.10%, with an Intersection over Union (IoU) colored range of [0-1]. This model enhances productivity by detecting and classifying date fruits based on surface quality. Koklu et al. (2021) classified date fruit types using three machine learning methods. 898 images were obtained, and 34 features were extracted. Logistic regression and artificial neural network methods were used, achieving 91.0% and 92.2% performance respectively. The stacking model combined these methods increased performance to 92.8%, proving successful application of machine learning methods. Safran et al. (2024) use 2358 images of four date palm species (Barhi, Sukkari, Ikhlas, and Saqi) and applied data augmentation techniques to increase the dataset’s diversity. They developed a lightweight CNN model called DPXception, which achieved the highest accuracy (92.9%) and F1-score (93%), with the lowest inference time (0.0513 seconds). The model was also used in an Android smartphone application to classify date palm species in real time. This is the first public dataset of date palm images and demonstrates a robust image-based species classification method. Chen et al. (2021) improve fruit maturity image recognition in IoT agriculture using a single shot multi-box detection algorithm and an image fusion algorithm based on improved Laplacian pyramid. Experiments show the proposed algorithm is feasible and effective. Xiao et al. (2023) review fruit detection and recognition using deep learning for automatic harvesting, addressing challenges like low-quality datasets, small targets, dense scenarios, multiple scales, and lightweight models. It proposes solutions and future development trends, aiming to improve accuracy, speed, robustness, and generalization while reducing complexity and cost. Song et al. (2020) presents an automatic detection and image recognition method for citrus diseases using the YOLO algorithm, which can detect and recognize diseases in real-time from captured images of citrus leaf diseases like Citrus Canker and Citrus Greening.

**Table 1:** The summary of recent works in fruit image recognition.

Authors	Method	Dataset	Pros	Cons	Performance
Sultana et al. (2024)	XAI-FruitNet (hybrid deep learning architecture)	Various datasets	Enhances feature discrimination, interpretability, and transparency; robust and trustworthy for agricultural needs.	N/A	Over 97% accuracy in fruit classification.
Marin et al (Marin and Radoi, 2022)	YOLOv3 (Convolutional Neural Networks)	Dataset made available	State-of-the-art object detection; dataset availability for further research.	Moderate classification accuracy.	Over 75% classification accuracy.
Huang et al. (2025)	Multi-model collaboration (YOLOv8 + Swin Transformer)	N/A	Improved detection and classification; optimized feature matching.	N/A	92.6% accuracy on 27 fruit categories; improved P, R, mAP50, and MAP50-95.
Rao Jerripothula et al. (2021)	Pre-trained neural networks and machine learning algorithms	Novel dataset named RipeRaw	High accuracy across agricultural, market, and automation perspectives.	N/A	96%, 94%, and 86% accuracies on RipeRaw dataset.
Chiagoziem et al (Ukwuoma et al., 2022)	Various deep learning methods	Fruit 360 dataset	Comprehensive review of methods and challenges; beginner-friendly.	Focuses on general fruit classification, not specific to date fruits.	(review paper).
Zarouit et al. (2024)	Image and video collection for detection and classification	Comprehensive dataset for date fruit maturity stages	Enhances automation in pre- and post-harvesting tasks.	N/A	(dataset paper).
Almutairi et al. (2024)	YOLOv8 (Deep Learning)	N/A	High precision and recall; enhances productivity in date fruit classification.	N/A	Mean recall: 0.99%; precision: 0.991%; IoU range: [0-1].
Koklu et al. (2021)	Logistic regression, artificial neural networks, and stacking model	898 images with 34 extracted features	High performance in date fruit classification; stacking model improves accuracy.	Limited to 898 images; feature extraction may not generalize to other datasets.	Logistic regression: 91.0%; ANN: 92.2%; stacking model: 92.8%.
Safran et al. (2024)	DPXception (lightweight CNN)	2358 images of four date palm species (Barhi, Sukkari, Ikhlas, Saqi)	High accuracy and F1-score; real-time classification on Android smartphones.	Limited to four date palm species.	92.9% accuracy; F1-score: 93%; inference time: 0.0513 seconds.
Chen et al. (2021)	Single shot multi-box detection + improved Laplacian pyramid image fusion	N/A	Feasible and effective for IoT-based fruit maturity recognition.	N/A	(algorithm feasibility demonstrated).
Xiao et al. (2023)	Review of deep learning for fruit detection and recognition	N/A	Addresses challenges and proposes solutions for future development.	Focuses on general fruit detection, not specific to date fruits.	(review paper).
Song et al. (2020)	YOLO algorithm	Captured images of citrus leaves	Real-time detection and recognition of citrus diseases.	Limited to citrus diseases; may not generalize to other fruits.	(real-time performance demonstrated).

From hybrid models like XAI-FruitNet to lightweight CNNs like DPXception, the field employs a wide range of techniques to address fruit recognition challenges. The high accuracy (e.g., 97% for XAI-FruitNet, 92.9% for DPXception) and real-time capabilities are common highlights.

## 3 Preliminaries

In this section, we describe the pre-trained architecture utilized in the proposed approach. The comparison includes two architectures designed to tackle classification tasks effectively. DenseNet121 with Squeeze-and-Excitation (SE) and YOLOv8n. Each model integrates specific components and methodologies to improve feature representation, model performance, and adaptability to custom datasets. DenseNet121 + Squeeze-and-Excitation is built on the DenseNet121 architecture pre-trained on the ImageNet dataset (Armağan et al., 2024). DenseNet121 leverages dense connections within its blocks, facilitating efficient gradient flow and promoting feature reuse. This model is further enhanced by the integration of a Squeeze-and-Excitation (SE) Attention block, which focuses on amplifying relevant features while suppressing irrelevant ones (Fırat and Üzen, 2024). This enhancement ensures that the model can concentrate on critical aspects of the data for improved classification performance (Alam et al., 2025). The architecture includes custom layers tailored for classification, such as GlobalAveragePooling2D and dense layers, allowing for effective feature aggregation and decision-making. The model is optimized using the Nadam optimizer with a learning rate of 0.0001, a batch size of 64, momentum set at 0.9, and trained over 50 epochs. These hyperparameter choices strike a balance between training efficiency and performance, making the model robust for various classification tasks (Hassan et al., 2023). YOLOv8n, on the other hand, is a lightweight object detection model pre-trained on a 15-class label date fruit dataset (Almutairi et al., 2024). This pre-trained model serves as a base for transfer learning and is fine-tuned on custom datasets to enhance its adaptability to domain-specific tasks. Unlike DenseNet121, YOLOv8n does not use a specific attention mechanism but relies on its efficient architecture to identify and classify objects (Ma et al., 2024a). The training process incorporates data augmentation techniques such as mixup (with a factor of 0.2), random flipping, and cropping, which enhance dataset diversity and improve the model’s robustness (Ma et al., 2024b). The Nadam optimizer is employed, with a higher learning rate of 0.01 to facilitate faster convergence. The model is trained for 70 epochs with a batch size of 16, balancing memory efficiency and processing speed. While the momentum is set at 0, the model’s architectural efficiency compensates for this by ensuring effective feature extraction and detection. Both models demonstrate unique strengths: DenseNet121 + SE excels in feature representation with its attention mechanism and robust architecture, while YOLOv8n offers a lightweight, adaptable solution optimized for domain-specific object detection tasks as shown in **Table 2**.

**Table 2:** Description of model settings used in this study.

Model	Description	Optimizer	Attention mechanism	Pre-trained?	The architecture	Hyperparameter values
DenseNet121 + Squeeze-and-Excitation	A DenseNet121 architecture pre-trained on ImageNet integrated with a Squeeze-and-Excitation (SE) Attention block to enhance feature representation.	Nadam	Squeeze-and-Excitation (SE) Attention block	Yes (ImageNet)	DenseNet121 with custom layers for classification, including GlobalAveragePooling2D and dense layers.	Learning rate: 0.0001, Batch Size: 64, Epochs: 50, Momentum: 0.9
YOLOv8n	A YOLOv8n model pre-trained on the 15 class label date fruit dataset, used as a base model for transfer learning with additional training on custom datasets.	Nadam	No	Yes	YOLO8	Learning rate: 0.01, Batch Size: 16, Epochs: 70, Momentum: 0 Data Augmentation: mixup (factor 0.2), random flipping, cropping.

### 3.1 DenseNet121 architecture

DenseNet (Densely Connected Convolutional Networks) (Huang et al., 2017; Bakr et al., 2022) is an architecture that significantly improves upon traditional convolutional neural networks (CNNs) by creating dense connections between layers. In DenseNet, each layer receives input from all previous layers, which allows for better feature propagation, reuse, and a reduction in the number of parameters compared to traditional CNNs (Elbedwehy et al., 2024). In the DenseNet121 architecture, there are 121 layers, structured into four dense blocks. The key component of DenseNet is the dense block, where each layer is connected to every other layer in a feedforward manner. Let $x_{i}\;$ represent the input to the  i −th layer in the dense block. In DenseNet, each subsequent layer $L_{i}$ receives not only the original input $x_{i}$ but also the outputs of all previous layers $\left\{L_{1},L_{2},\ldots ,L_{i-1}\right\}$ (Yu et al., 2021). Mathematically, the output of the  i −th layer is computed as in Equation 1:


(1) $$L_{i}=F_{i}([x_{o},x_{1},\ldots ,x_{i-1}])$$

where:

-
$\left[x_{0},x_{1},\ldots ,x_{i-1}\right]$ denotes the concatenation of all previous layer outputs.
-
$F_{i}$ is the operation performed by the  i −th layer, which typically includes a batch normalization (BN), ReLU activation, and convolution operation.

By using dense connections, DenseNet enables feature reuse, which leads to more efficient learning, especially in deep networks, and mitigates the vanishing gradient problem by improving gradient flow (Saber et al., 2025). This architecture is often used for tasks that require deep feature extraction with fewer parameters.

### 3.2 Squeeze-and-Excitation Attention block

The block Squeeze-and-Excitation (SE) Attention is a mechanism designed to improve the representational power of a model by adaptively recalibrating channel-wise feature responses. It enhances the model’s ability to focus on informative features and suppress less relevant ones, which is particularly useful for fine-grained classification tasks. The SE block operates in two main steps: squeeze and excitation (Zeng et al., 2025).

#### 3.2.1 Squeeze: global average pooling

In the squeeze step, the feature map F  with dimensions H ×W ×C  (height H , width W, and channels C) is passed through a global average pooling (GAP) operation to generate a channel descriptor (Jin et al., 2022). This operation computes the average value of each channel across the spatial dimensions (height and width), resulting in a 1D vector  z  of length  C as in Equation 2.


(2) $$z_{c}=\frac{1}{H\times W}\sum_{h=1}^{H}\sum_{w=1}^{W}F_{h,w,c}$$

where:

-
$F_{h,w,c}$ is the value of the feature map at position ((h,w)) for channel ( c ) .
-
$z_{c}$ is the average pooled value for the c−th channel.
- The output of this step is a channel-wise global descriptor vector $z=[z_{1},z_{2},\ldots ,z_{C}])of\;size\;C\times 1$ .

#### 3.2.2 Excitation: fully connected layers and sigmoid activation

The excitation step recalibrates the channel-wise features based on the global context provided by the vector z . The process begins by passing z through two fully connected (FC) layers with ReLU activation in the first layer, followed by a sigmoid activation in the second layer. The two FC layers aim to learn the importance of each channel (Zhu et al., 2021). The excitation step can be described by the following Equation 3.


(3) $$s=\sigma (W_{2}\delta (W_{1}z+b_{1})+b_{2})$$

where: z is the input channel descriptor from the squeeze step.

-
$W_{1}and\;W_{2}$ are weight matrices for the two fully connected layers.
-
$b_{1}and\;b_{2}$ are the corresponding biases.
-
δ is the ReLU activation function.
-
σ is the sigmoid activation function.
-
s  is the output scalar channel importance vector, which has the same dimension as (z),i.e.,(C×1) .

#### 3.2.3 Recalibration

Finally, the recalibration step applies the learned importance values (from the excitation step) to the input feature map ( F ) . This is done by scaling each channel of ( F ) by the corresponding scalar in ( s ) (Mulindwa and Du, 2023). Mathematically, the recalibration is performed as in Equation 4.


(4) $$\hat{F_{h,w,c}}=F_{h,w,c}\cdot s_{c}$$

where:

-
$\hat{F_{h,w,c}}\;$ is the recalibrated feature map.
-
$s_{c}$ is the scalar value for channel  c obtained from the excitation step.

By scaling the feature map, the SE block enables the network to emphasize the most informative channels and suppress less useful ones, improving the performance of the network in tasks requiring fine-grained feature distinctions. The DenseNet121 architecture and the Squeeze-and-Excitation (SE) attention block provide complementary advantages in deep learning models. DenseNet improves feature propagation and reuse through dense connections, while SE attention blocks recalibrate feature maps at the channel level, allowing the network to focus on the most important features. These techniques are powerful when used together, particularly in tasks like image classification, where fine-grained attention to feature details is crucial (Deng et al., 2020; Peng et al., 2023; Stergiou and Poppe, 2023).

### 3.3 YOLOv8n

YOLOv8n is a lightweight and efficient object detection model designed for real-time applications, particularly on resource-constrained devices. It uses a slimmed-down CSP-based backbone for feature extraction, a Feature Pyramid Network (FPN) and Path Aggregation Network (PAN) in the neck for multi-scale feature fusion, and an anchor-free head for simplified and efficient bounding box prediction (Fan and Liu, 2024). The model leverages advanced data augmentation techniques like mixup, mosaic, random flipping, and cropping to enhance robustness and generalization (Kaur et al., 2021).

Optimized with an Nadam optimizer and a composite loss function combining bounding box, classification, and objectness losses, YOLOv8n achieves high accuracy with minimal computational overhead. Its hyperparameters, including a learning rate of 0.01, batch size of 16, and 70 training epochs, ensure efficient convergence. These features make YOLOv8n suitable for real-time applications in robotics, autonomous systems, and edge devices. YOLOv8n uses a composite loss function to optimize object detection performance. The total loss L is defined as in Equation 5.


(5) $$L=L_{box}+L_{cls}+L_{obj}$$

where:

-
$L_{box}$ : Bounding box regression loss (e.g., CIoU or DIoU loss),
-
$L_{cls}$ : Classification loss (e.g., binary cross-entropy for each class),
-
$L_{obj}$ : Objectness score loss.

The loss function is designed to balance the accuracy of bounding box localization, object detection, and class prediction (Khow et al., 2024).

## 4 The proposed method

This paper presents two deep learning models designed for image classification and object detection, leveraging state-of-the-art architectures to achieve high accuracy and robustness. The first model integrates a DenseNet121 architecture pre-trained on ImageNet with a Squeeze-and-Excitation (SE) Attention block to enhance feature representation and improve model focus on critical details. By employing data augmentation techniques, such as rotation, shifting, shearing, zooming, and flipping, the model improves generalization and reduces overfitting. The architecture is further refined with custom layers, including GlobalAveragePooling2D and dense layers, for optimal classification performance. The model is trained using the Nadam optimizer with a learning rate of 0.0001 and evaluated using various metrics such as accuracy, precision, recall, F1 score, and ROC-AUC.

The second model employs a YOLOv8n architecture, pre-trained on the fruit dataset, and fine-tuned for object detection on a custom dataset. The model processes images resized to 640 pixels and applies advanced data augmentation techniques, including mixup, random flipping, and cropping, to enhance generalization. It is trained for 50 epochs with a batch size of 16 and an initial learning rate of 0.01. The training pipeline includes a composite loss function optimizing bounding box regression, classification, and objectness scores. After training, the model is evaluated on a validation set and deployed for real-time inference on images and videos, making it suitable for real-world applications in automated detection and classification tasks.

### 4.1 The first proposed model

This paper proposes a novel image classification approach by integrating a DenseNet121 model pre-trained on ImageNet with a Squeeze-and-Excitation (SE) Attention block. The integration of the SE Attention block enhances feature representation by allowing the model to focus on the most relevant features, improving classification performance. To ensure robust generalization and mitigate overfitting, various data augmentation techniques are applied, diversifying the training data and making the model more adaptable to unseen samples.

#### 4.1.1 Data preparation and augmentation

To effectively train the model, the dataset is first organized into a structured folder format, categorizing images based on their respective classes. Data augmentation is performed using the ImageDataGenerator function, incorporating transformations such as rotation, width and height shifts, shearing, zooming, and flipping. These transformations enhance dataset diversity and improve model robustness. The dataset is then divided into training and validation subsets, with 80% allocated for training and 20% for validation, ensuring an effective balance for model learning and performance assessment.

#### 4.1.2 Model definition

The DenseNet121 architecture, pre-trained on the ImageNet dataset, is used as the backbone of the classification model (Chutia et al., 2024). To enhance feature extraction capabilities, a Squeeze-and-Excitation (SE) Attention block is incorporated. This mechanism adaptively recalibrates feature maps by modeling interdependencies between channels, allowing the model to focus more on essential features. Custom layers are added for classification, including a GlobalAveragePooling2D layer to reduce spatial dimensions and dense layers to refine feature representation and optimize classification accuracy.

#### 4.1.3 Model compilation and training

The model is compiled using the Nadam optimizer, known for its adaptive learning rate capabilities, with an initial learning rate of 0.0001. The training process is conducted for 20 epochs, where model performance is continuously monitored on the validation dataset. This ensures that the model achieves optimal convergence while avoiding overfitting. The adaptive learning mechanism of Nadam facilitates efficient parameter updates, leading to better generalization.

#### 4.1.4 Performance evaluation

Once trained, the model is evaluated using multiple performance metrics to assess its effectiveness in image classification. These metrics include accuracy, precision, recall, F1-score, and ROC-AUC, providing a comprehensive evaluation of classification performance. These measures ensure that the model not only performs well on the training data but also generalizes effectively to unseen data, making it a reliable approach for date fruit image classification.

#### 4.1.5 Model architecture design

The DenseNet architecture is distinguished by its dense connectivity pattern, where each layer receives direct input from all preceding layers, facilitating efficient feature reuse. To further enhance the model’s ability to focus on informative features, integrating attention blocks within the DenseNet framework (Liu and Zeng, 2018). Mathematically, the output $\left(X_{k}\right)$ of the ( k ) -th dense block in DenseNet is computed as shown in Equation 6.


(6) $$X_{k}=X_{k-1}⊕G_{k}(X_{k-1})$$

where $\left(G_{k}\right)$ represents the composite function of convolutional, batch normalization, and Rectified Linear Unit (ReLU) operations within the ( k ) -th dense block, and ( ⊕) denotes concatenation. To incorporate attention mechanisms, introducing attention blocks $\left(B_{k}\right)$ after each dense block, generating attended features $\left(B_{k}\right)$ as described in Equation 7.


(7) $$B_{k}=B_{k}(X_{k})$$

The attention block $\left(B_{k}\right)$ typically consists of a combination of operations, including global average pooling, linear transformations, and activation functions. The attended features are then combined with the output of the dense block to form the input for the next layer, as expressed in Equation 8.


(8) $$X_{k+1}=B_{k}⊕G_{k+1}(B_{k})$$

This modification enhances the model’s ability to emphasize relevant features, adapting to the specific characteristics of the input data. The attention mechanisms contribute to improved discriminative power, making the DenseNet architecture more effective at capturing nuanced patterns within the input sensor data (Zhou et al., 2022; Mujahid et al., 2023).

#### 4.1.6 Hyperparameter tuning and evaluation

In this section, focusing on optimizing key parameters to enhance the performance of the proposed DenseNet model with attention mechanisms. The Nadam optimizer, which combines Nesterov accelerated gradient (NAG) and Nadam optimization techniques, is employed to efficiently update model parameters during training (Abdulkadirov et al., 2023; Reyad et al., 2023). The update rule for the model parameters ( ϕ) using Nadam is expressed in Equations 9–13.


(9) $$u_{t}=\gamma_{1}\cdot u_{t-1}+(1-\gamma_{1})\cdot \nabla L(\phi_{t})$$

(10) $$w_{t}=\gamma_{2}\cdot w_{t-1}+(1-\gamma_{2})\cdot {\left(\nabla L(\phi_{t})\right)}^{2}$$

(11) $$\hat{u_{t}}=\frac{u_{t}}{1-\gamma_{1}^{t}}$$

(12) $$\hat{w_{t}}=\frac{w_{t}}{1-\gamma_{2}^{t}}$$

(13) $${\text{ϕ}}_{t+1}={\text{ϕ}}_{t}-\frac{\text{α}}{\sqrt{\hat{w_{t}}}+\text{δ}}\cdot (\gamma_{1}\cdot \hat{u_{t}}+\frac{\left(1-\gamma_{1})\cdot \text{∇}L({\text{ϕ}}_{t}\right)}{1-\gamma_{1}^{t}})$$

where α is the learning rate, $\nabla L(\phi_{t})$ is the gradient of the loss with respect to the parameters, $\gamma_{1}$ and $\gamma_{2}$ are exponential decay rates for the moment estimates, and δ is a small constant to prevent division by zero. The batch size S is another critical hyperparameter that influences the number of samples used in each iteration (Bhat and Birajdar, 2023). A smaller batch size may introduce more noise but can lead to faster convergence, while a larger batch size provides a smoother gradient but requires more computational resources.

**Figure 1:**
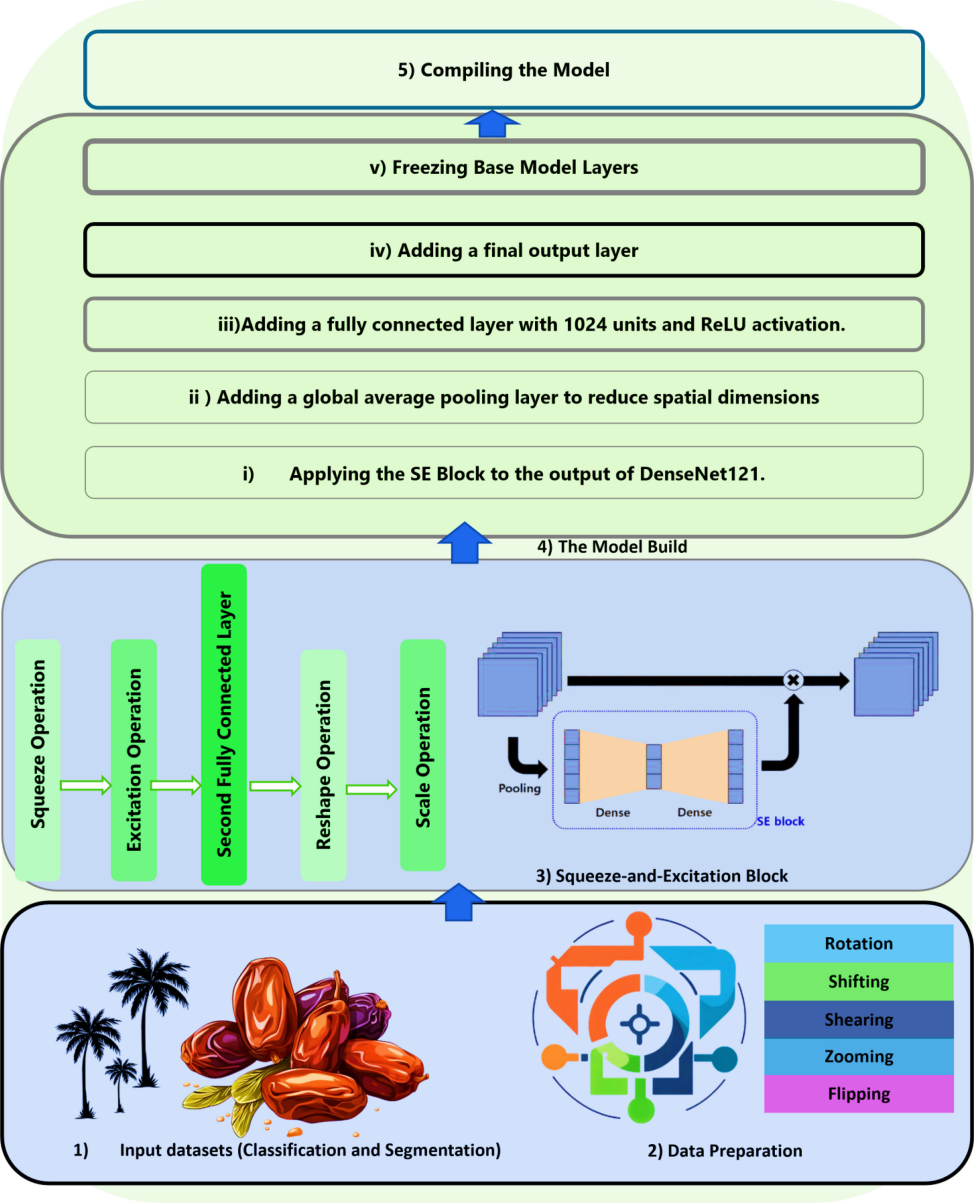
The structure of the proposed DensNet121-SE model.

For the attention mechanisms, the attention weights (α) can be fine-tuned to balance the contribution of attended features in the model output (Bi et al., 2023). The attention block’s mathematical representation is given in Equation 14:


(14) $$B_{k}=\sigma (W_{\alpha }\cdot \text{GAP}(G_{k}(X_{k})))$$

where (σ) is the activation function, $\left(W_{\alpha }\right)$ represents the attention weights, and (GAP) denotes the global average pooling operation (Asif et al., 2023). By employing the Nadam optimization algorithm, which combines the Nesterov accelerated gradient and Nadam optimization techniques, the model benefits from efficient parameter updates during training. This ensures that the attention weights are optimized to effectively emphasize the most relevant features, enhancing the model’s performance. The proposed DensNet121+SE model steps are shown in detail in **Figure 1**.

The figure outlines a structured process for building and compiling a deep learning model based on the DenseNet121 architecture, incorporating a Squeeze-and-Excitation (SE) Block to enhance feature representation. The process begins with preparing input datasets for classification and segmentation, ensuring they are properly loaded and preprocessed. Data preparation involves normalization, augmentation, and splitting the dataset into training, validation, and test sets to enhance generalization. The SE Block is then introduced to improve the network’s representational power by modeling interdependencies between channels through global average pooling and a self-gating mechanism. The model is constructed by applying the SE Block to the output of DenseNet121, followed by a global average pooling layer to reduce spatial dimensions, a fully connected layer with 1024 units and ReLU activation, and a output layer for predictions. Compilation involves defining the optimizer, loss function, and evaluation metrics, setting up the learning process. To fine-tune the model, the base layers of DenseNet121 are frozen, preserving pre-trained features while allowing the newly added layers to learn task-specific representations.

### 4.2 The second proposed model

The second model utilizes the YOLOv8n architecture, a state-of-the-art object detection framework, to classify date fruit images. The first step in this approach involves data preprocessing, where images are resized to a standard dimension of 640×640 pixels to ensure consistency across the dataset. Various data augmentation techniques, including mixup, random flipping, and cropping, are applied to improve model generalization, reduce overfitting, and enhance robustness against variations in lighting, orientation, and background noise. Once the data is prepared, the model initialization phase begins by loading a pre-trained YOLOv8n model (yolov8n.pt). This model, originally trained on the COCO dataset, serves as a strong foundation for transfer learning, enabling the network to adapt to the specific characteristics of the date fruit dataset with minimal training from scratch. The training pipeline is then set up with key hyperparameters, including a batch size of 16, which balances computational efficiency and model convergence, and a learning rate of 0.01, which governs the step size during weight updates. The model is trained for 50 epochs, allowing sufficient time for convergence while preventing overfitting. Additionally, advanced data augmentation techniques such as mixup (with a factor of 0.2) are employed to increase dataset diversity and enhance the model’s ability to generalize to unseen data. During validation and evaluation, the trained model is tested against a reserved validation set to measure its performance. Various performance metrics, including accuracy, precision, recall, and mean Average Precision (mAP), are used to assess the effectiveness of the model. After training and evaluation, the model is saved for future deployment. The trained model can then be used for real-time inference, where it can classify new images or videos by loading the saved weights and running the detection pipeline. This ensures that the model is not only effective in an experimental setting but also practical for real-world applications, such as automated fruit sorting or quality assessment. To mitigate overfitting given the small dataset size, several strategies were employed. Data augmentation techniques, including random flipping, rotation, and scaling for DenseNet121+SE, and mixup (factor 0.2), random cropping, and flipping for YOLOv8n, were applied to enhance generalization. Regularization techniques such as dropout layers and L2 regularization (weight decay) were used to control model complexity. Transfer learning played a crucial role, with DenseNet121 pre-trained on ImageNet and YOLOv8n pre-trained on a 15-class date fruit dataset, leveraging prior knowledge to improve performance. Early stopping was implemented to halt training when validation loss ceased to improve, preventing unnecessary overfitting. Additionally, hyperparameter tuning was carefully performed, with a learning rate of 0.0001 for DenseNet and 0.01 for YOLOv8n, while batch normalization stabilized training. These combined strategies ensured robust generalization and reliable model performance beyond the training dataset.

## 5 Experimental and results

### 5.1 Dataset characteristics

The dataset comprises 1,658 high-quality JPG images, depicting Saudi Arabian date fruit types: Ajwa, Galaxy, Medjool, Meneifi, Nabtat Ali, Rutab, Shaishe, Sokari, and Sugaey. The dataset and improve generalization, data augmentation techniques such as rotation, flipping, zooming, and cropping are applied and the resulting dataset are 1868 images. The dataset is divided into training, validation, and test sets: Training: 80% of the data (1,494 images), Validation: 10% of the data (187 images), Test: 10% of the data (187 images). Class Imbalance: Some classes (e.g., Sokari: 264, Meneifi: 232) have more images than others (e.g., allig: 104, deglet_nour: 106) as shown in **Table 3**. Data augmentation is used to mitigate class imbalance. Date fruit classification using segmented labels categorizes date fruits into different distinct classes based on physical and visual characteristics to uses advanced deep learning techniques like CNNs or transformer-based models to accurately identify and differentiate between labeled classes.

**Table 3:** The number of images representing the date fruits types after augmentation.

Class name	Number of images
Ajwa	175
Galaxy	190
Medjool	135
Meneifi	232
Nabtat Ali	177
Rutab	146
Shaishe	171
Sokari	264
Sugaey	168
allig	104
deglet_nour	106
Total	1868

### 5.2 Results and analysis

To assess the performance of the proposed framework, conduct a series of experiments. These experiments were executed on a computer equipped with a 3 GHz Intel Core i7 processor, 8 GB of RAM, and a 64-bit Windows 10 operating system. The implementation was carried out using the Python programming language, ensuring efficient execution and reproducibility of the experimental setup.

### 5.3 Pre-trained models

#### 5.3.1 The First model based DenseNet121-SE

**Figure 2:**
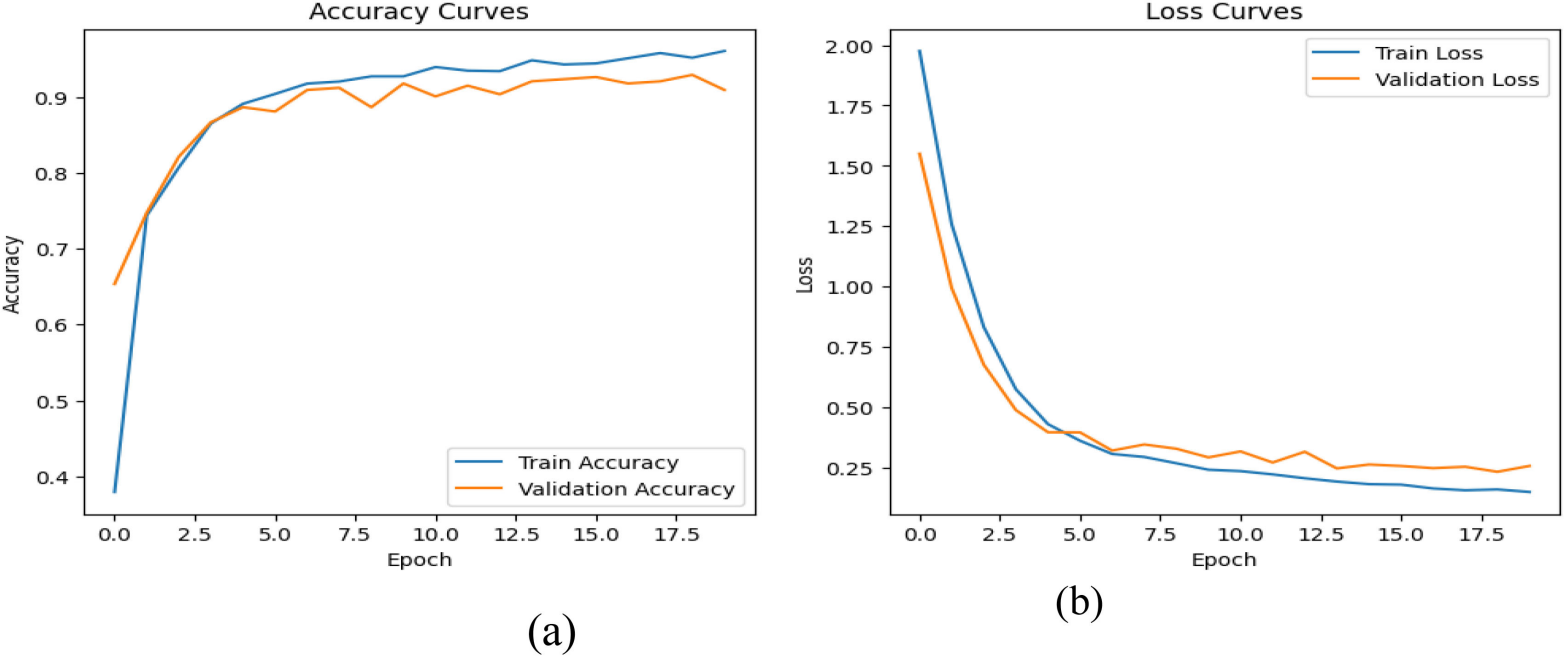
The learning curves for DensNet121-SE model **(a)** The training and validation accuracy, **(b)** The training and validation loss.

Analyzing the performance of the DenseNet121 architecture integrated with a Squeeze-and-Excitation (SE) block by closely examining its learning curves and conducting a series of rigorous experiments. The learning curves provide a comprehensive visual representation of the model’s progress over epochs, highlighting convergence trends and adaptability. **Figure 2** illustrates these patterns specifically for the Nadam optimizer. Through a carefully structured set of experiments, adjusting SE block parameters and assessing their impact on the model’s responsiveness across different datasets and tasks. By monitoring key metrics such as accuracy, loss, and convergence rates, aiming to uncover the intricate relationship between the SE-enhanced DenseNet121 architecture and its ability to extract meaningful features effectively.

**Figure 3:**
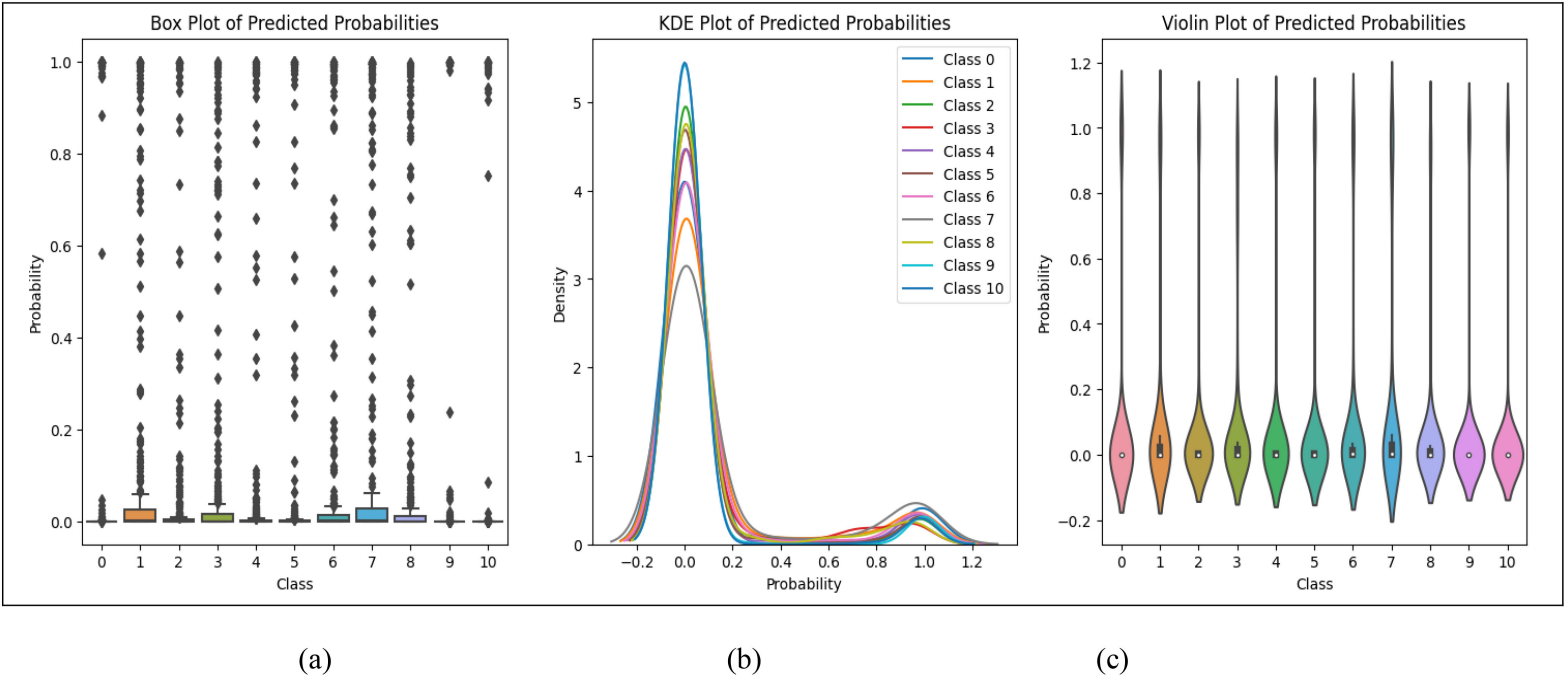
The statistical analysis **(a)** Box Plot, **(b)** KDE plot and **(c)** Violin plot of predicted probabilities for dataset classification.

**Figure 3** illustrates the statistical distribution of predicted probabilities using three visualization techniques: (a) Box Plot, (b) Kernel Density Estimation (KDE) Plot, and (c) Violin Plot. The Box Plot highlights the spread, median, and potential outliers in the predictions, revealing variations in model confidence. The KDE Plot provides a smoothed probability density function, showing where predictions are concentrated and indicating confidence levels. The Violin Plot combines both, offering a detailed view of probability distribution and density across different classes. These analyses help assess model performance, identify uncertainties, and refine classification strategies.

**Figure 4:**
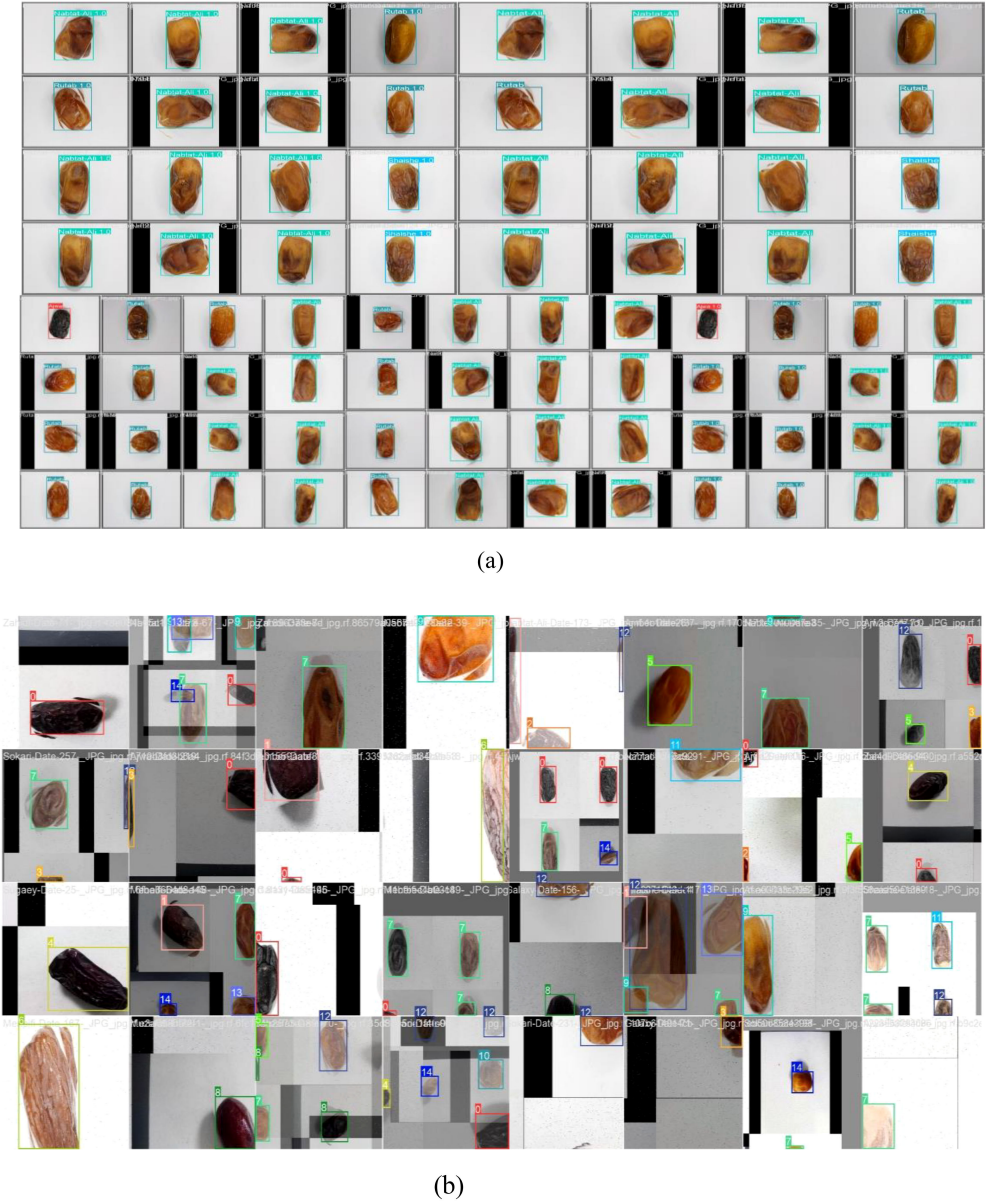
Sample images for date fruit segmentation – **(a)** testing set, **(b)** training set.

**Figure 5:**
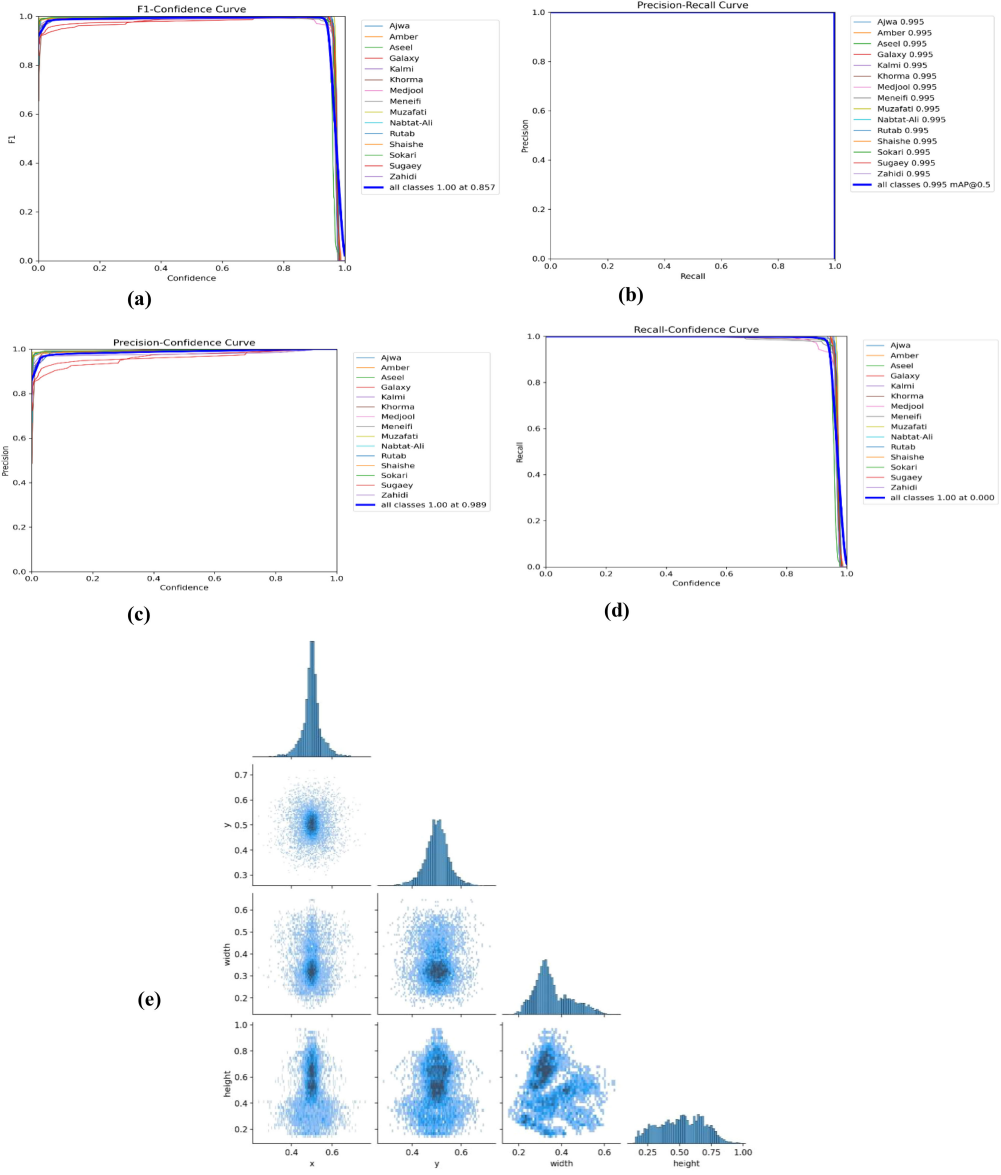
Comprehensive performance evaluation and feature distribution analysis. **(a)** F1-Confidence Curve, **(b)** Precision-Recall Curve, **(c)** Precision-Confidence Curve, **(d)** Recall-Confidence Curve, and **(e)** Pairplot Visualization.

**Figure 6:**
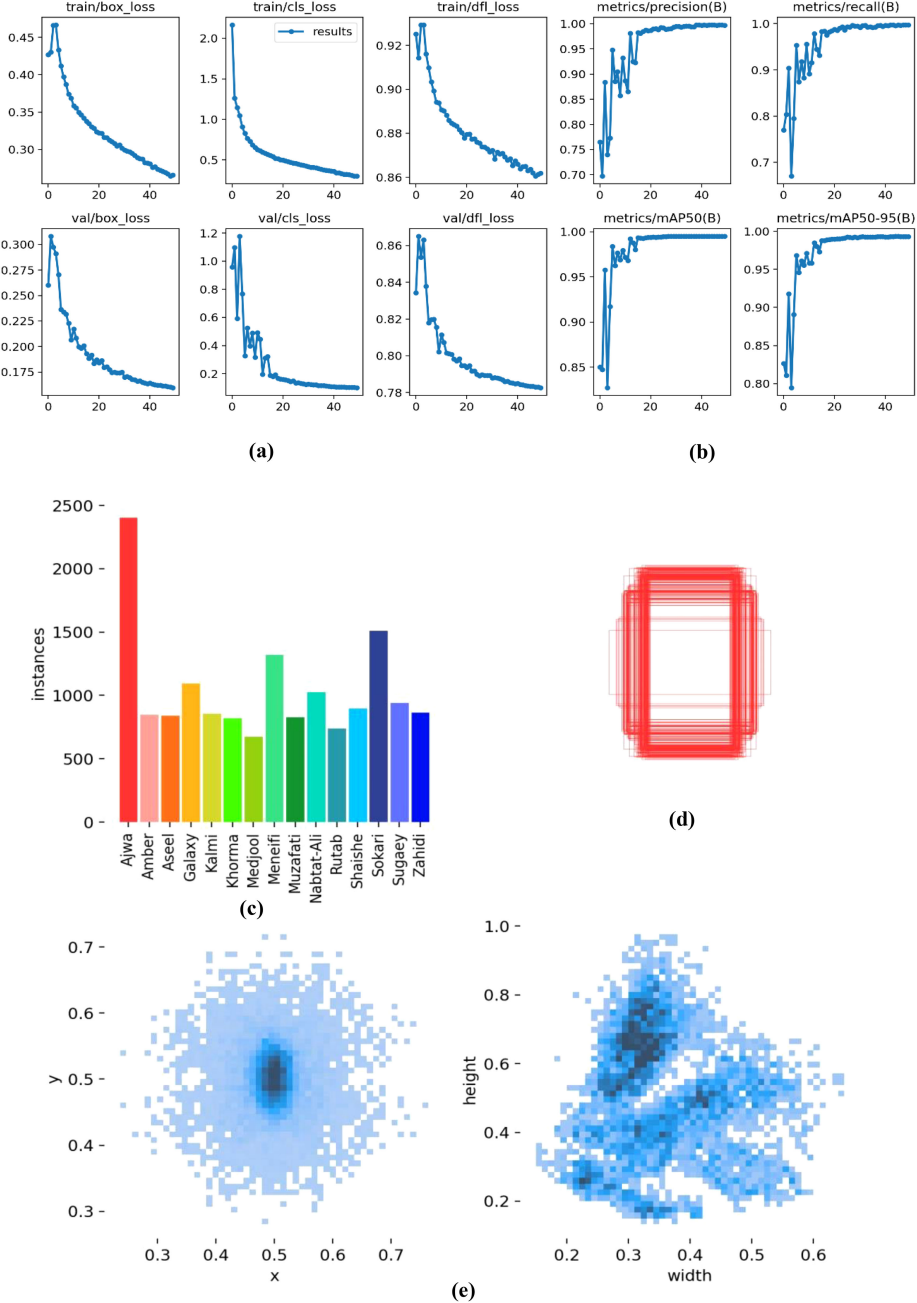
Model training and evaluation visualizations. **(a)** Training and Validation Loss Metrics, **(b)** Precision, Recall, and mAP Metrics, **(c)** Instance Distribution Bar Chart, **(d)** Bounding Box Visualization, and **(e)** Density Plots.

**Figure 4** displays a collection of tested and trained classified date fruit images, each labeled with their respective categories using a bounding box and text annotation. The dataset includes various date types, such as “Nabtat-Ali,” “Rutab,” “Shaishe,” and “Ajwa,” showcasing different textures, shapes, and colors. The images appear to be part of an object detection or classification task to automate the identification process. **Figure 5** presents a comprehensive set of performance evaluation plots and a pairplot visualization, providing insights into the model’s classification capabilities. The F1-Confidence Curve illustrates the F1 scores across different confidence thresholds for individual classes and the model performance. The Precision-Recall Curve highlights the trade-off between precision and recall for each class, with the mean average precision (mAP@0.5) serving as a key performance indicator. The Precision-Confidence Curve showcases how precision varies with confidence levels, while the Recall-Confidence Curve represents the recall as a function of confidence, providing a comparative analysis across different classes. Additionally, the Pairplot Visualization presents scatter plots and histograms of spatial features (x, y, width, height), revealing their distributions and interdependencies. **Figure 6** presents key visualizations related to model training and evaluation, providing insights into performance metrics and dataset characteristics.

#### 5.3.2 The second model based YOLOv8n

**Figure 7:**
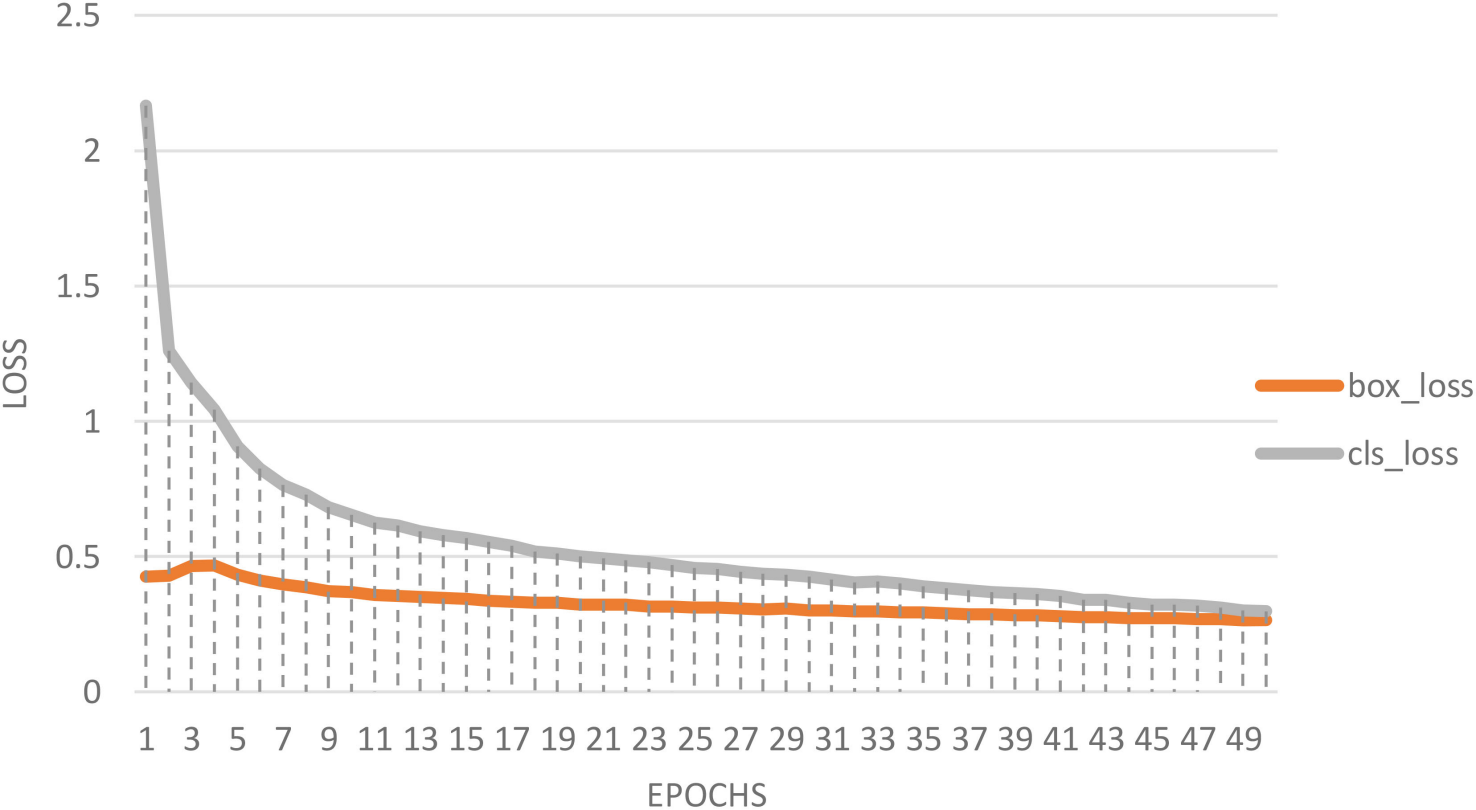
The box loss and cls loss of the proposed model based YOLOv8n.

Presenting the classification of date fruit images based on YOLOv8n. **Figure 7** represents the loss curve plot illustrating the training progression of a YOLOv8n-based classification model over 50 epochs, depicting box loss and classification loss. Initially, classification loss starts above 2.0 and box loss around 0.4–0.5, indicating significant errors. During the first 10 epochs, classification loss decreases rapidly, while box loss declines more gradually. Beyond 20 epochs, both losses stabilize, showing steady convergence. By epoch 50, both losses reach relatively low values, suggesting improved model accuracy and robustness. The narrowing gap between box and classification losses indicates balanced learning of localization and classification tasks. This visualization is essential for evaluating model performance, ensuring proper convergence, and identifying areas for optimization, such as fine-tuning hyperparameters like learning rate, batch size, or data augmentation strategies.

**Figure 8:**
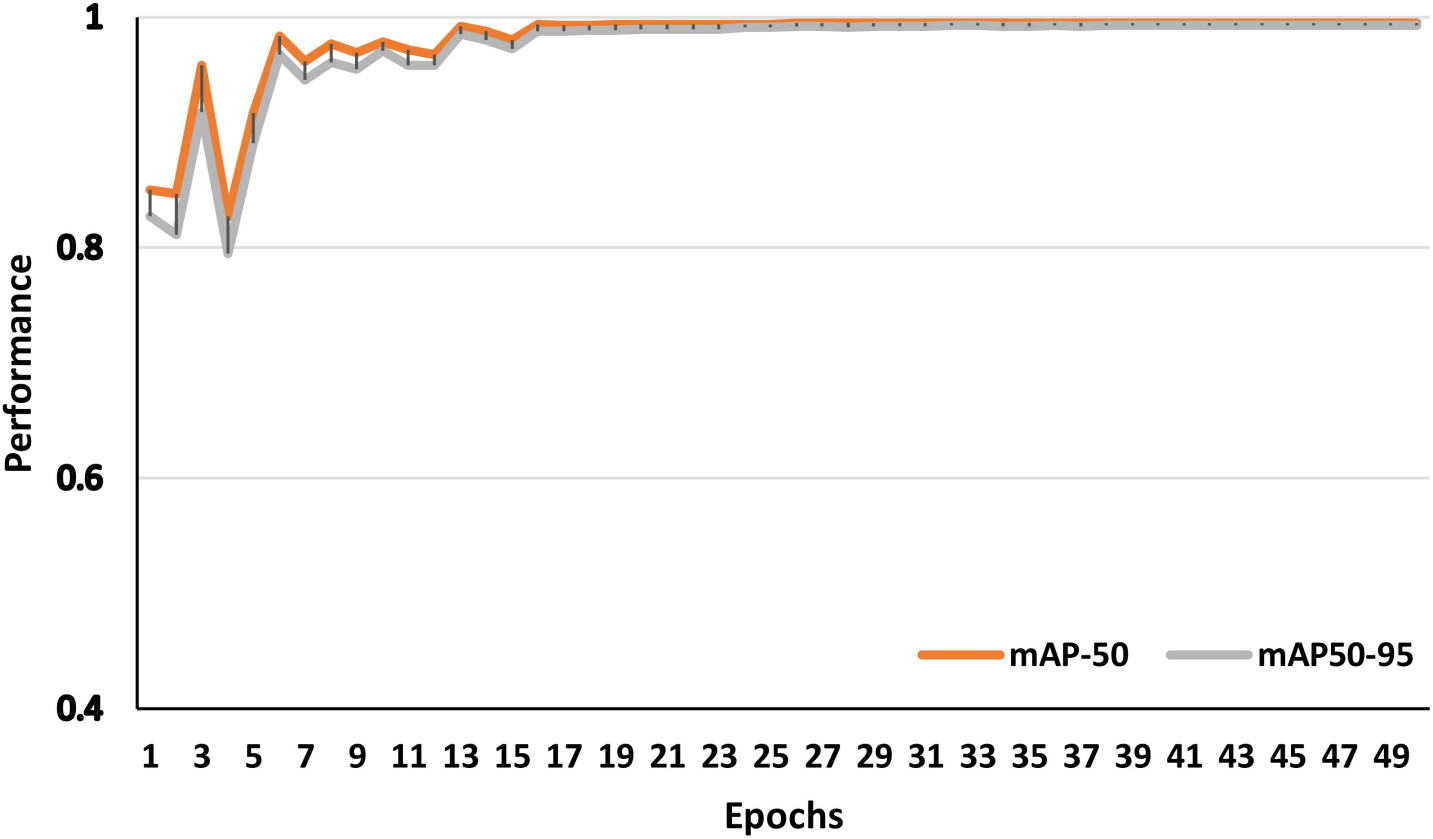
The mAP-50 and mAP-95 performance of the proposed model based YOLOv8n.

**Figure 9:**
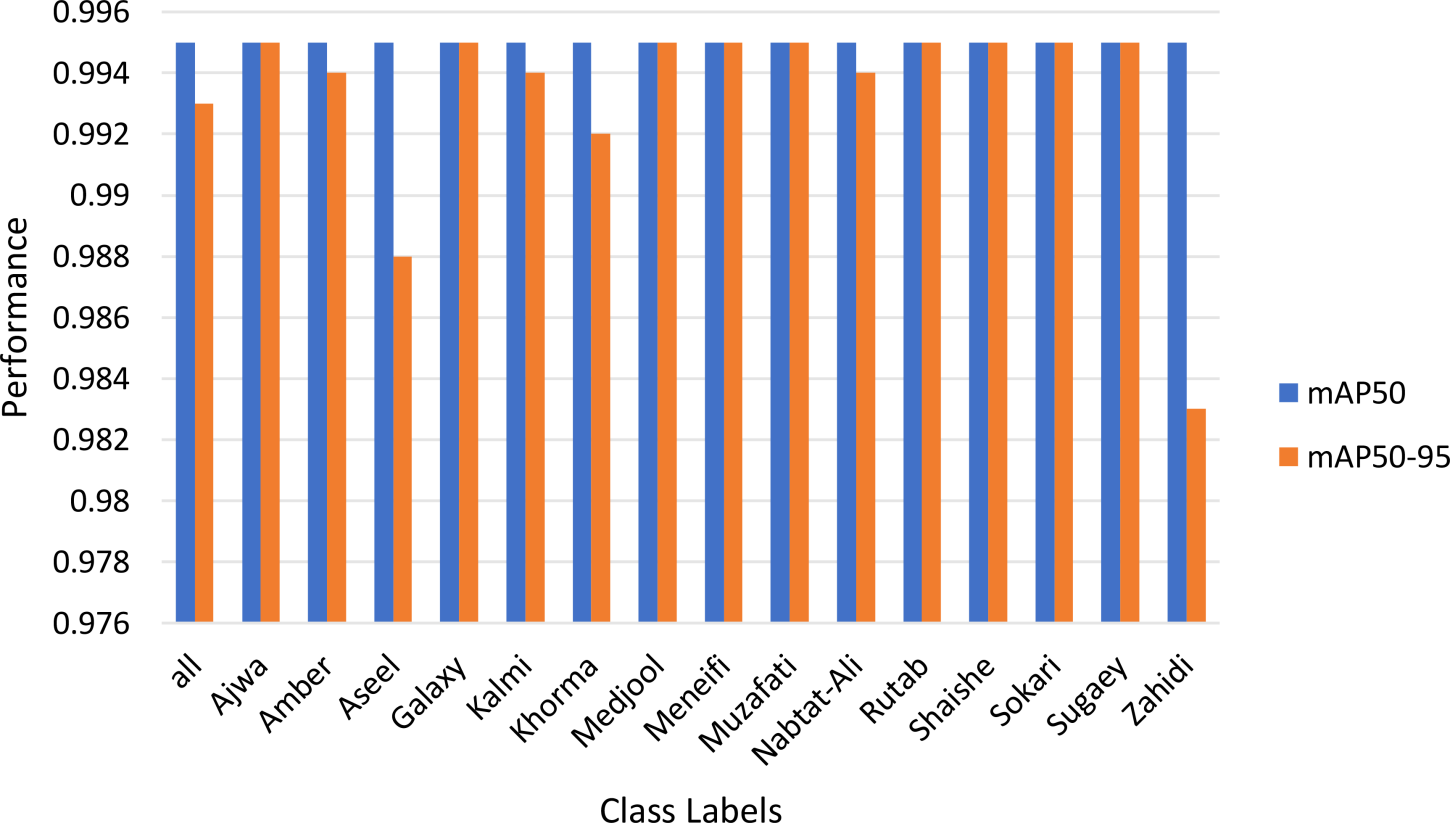
The mAP-50 and mAP50–95 by Class using YOLOv8n.

**Figure 8** illustrates the model’s performance of over 50 training epochs using the YOLOv8n framework, represented by two key metrics: mAP-50 (mean Average Precision at threshold 0.5) and mAP50-95 (mean Average Precision averaged across thresholds from 0.5 to 0.95). At the initial epochs, both metrics fluctuate, indicating an unstable learning phase. However, from around epoch 10 onward, both mAP-50 and mAP50–95 stabilize and converge near 1.0, signifying high model accuracy. The mAP-50 measures precision at a single Intersection over Union (IoU) threshold (0.5), which means it evaluates how well the model detects objects with at least 50% overlap between the predicted and ground truth bounding boxes. In contrast, mAP50–95 is a more stringent metric, averaging precision over multiple IoU thresholds (0.50, 0.55, 0.95), providing a more comprehensive assessment of the model’s detection performance across varying levels of localization accuracy. In this case, the minimal gap between the two metrics suggests that the model performs consistently well across different IoU thresholds, indicating strong localization precision and robustness in detecting date fruit images. **Figure 9** investigates the mAP-50 and mAP50–95 for different class labels utilized in this work.

**Figure 10:**
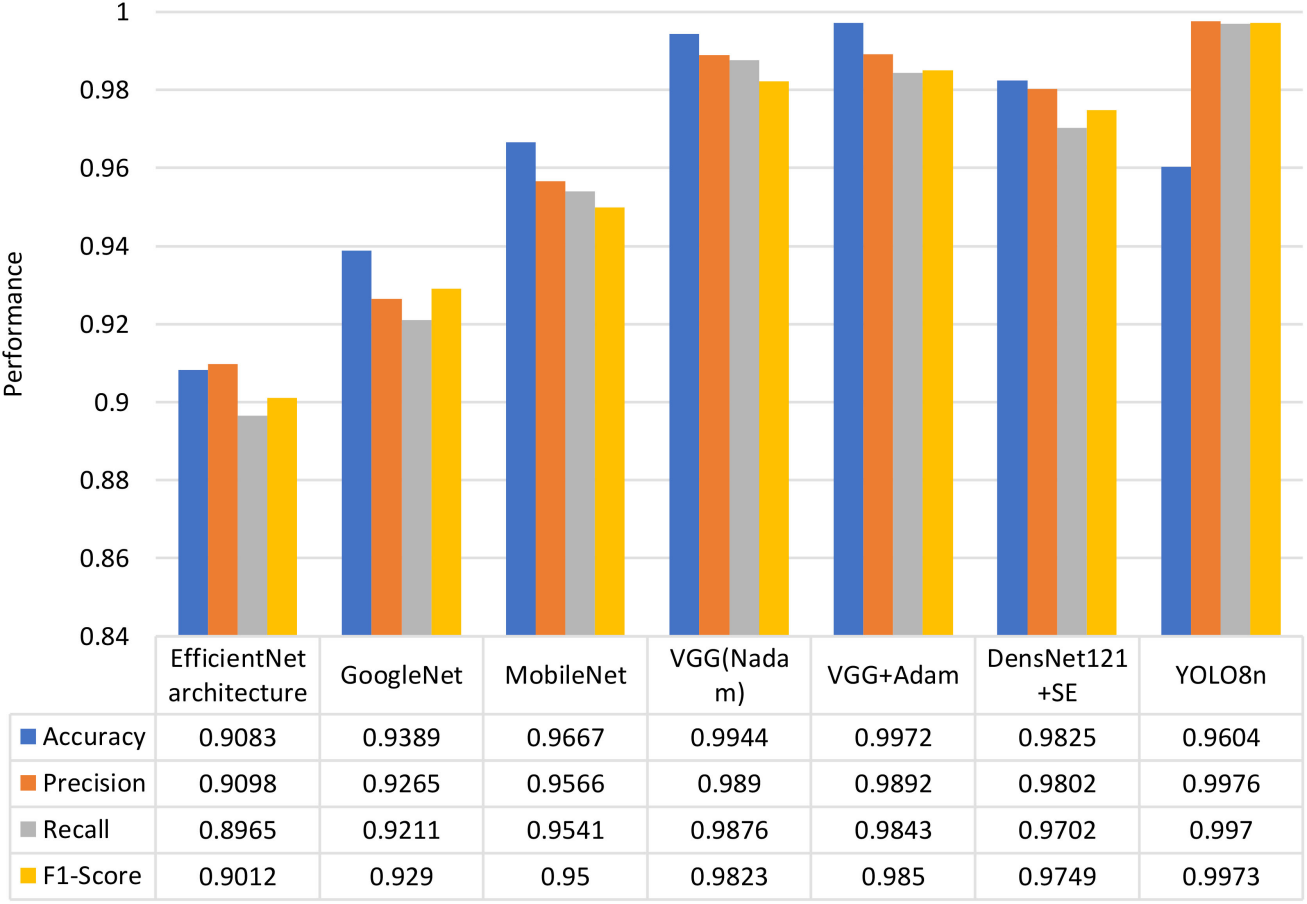
The comparative analysis between most recent DL approaches and the proposed models.

The model exhibits high precision (0.9976) and recall (0.9970), ensuring exceptional detection accuracy. The mAP50 remains consistently high (0.995) across most classes, with minor variations for Aseel (0.98781) and Zahidi (0.9834). The model processes data efficiently, with an inference time of 3.60 seconds and postprocessing time of 2.14 seconds, making it suitable for real-time applications. The fitness score of 0.9935 highlights its strong performance. Additionally, both training and validation losses decrease significantly over epochs, reflecting effective learning. The training accuracy improves from 0.3793 to 0.9604, while validation accuracy increases from 0.6534 to 0.9290. After Epoch 10, the model stabilizes, with only minor fluctuations in validation accuracy and loss. The training time per epoch ranges between 452s and 542s, averaging ~480s, demonstrating efficient training dynamics. The comparison between the proposed methods with the deep leaning approaches Efficient Net, Google Net, VGG (Nadam), VGG (Adam) are shown in **Figure 10**.

## 6 Discussion

This paper aims to address several key challenges in the automation of fruit classification and quality control in agriculture. While the proposed model demonstrates high performance, it is important to acknowledge that its robustness could be further enhanced with a larger and more diverse dataset. The current dataset, although augmented, may not fully capture the variability present in real-world farm environments, such as changing lighting conditions, background noise, and image quality variations. These factors could introduce biases into the model, which might affect its generalizability. Furthermore, the dataset primarily focuses on date fruits, and although our model performs well for these, its application to other fruit types or cultivars may require further validation with a more varied and expansive dataset. The integration of the DenseNet121 architecture with the Squeeze-and-Excitation (SE) attention mechanism is designed to improve the model’s ability to focus on critical image features, which enhances classification performance. However, deploying this model in real-world agricultural settings still presents challenges, particularly when considering the complexities of varying lighting, occlusions, and cluttered backgrounds that often arise in farm environments. For practical applications, real-time processing of large volumes of image data is essential, and this can be a significant hurdle in areas with limited computational resources. To address these concerns by presenting YOLOv8n as an efficient solution for real-time classification. This model, with its high accuracy and lightweight design, offers a viable option for deployment in automated agricultural systems. The use of YOLOv8n also makes the approach more applicable to real-world settings, particularly when dealing with a broader range of fruit varieties. Looking forward, several areas for improvement and future work emerge. Expanding the dataset to include a wider range of fruit varieties and real-world conditions is a critical next step. Such an expansion would enhance the generalizability of the model and its ability to classify a diverse set of fruits under varying environmental conditions. Testing the model on fruits with similar visual characteristics could further demonstrate its versatility. Additionally, exploring semi-supervised or unsupervised learning techniques could help mitigate the challenges posed by limited labeled data in agricultural contexts. While the current model performs well, further optimization may be needed to reduce its computational demands for use in low-resource environments. Future work could focus on model compression or lightweight architectures that maintain performance while improving efficiency. Integrating the model into larger agricultural management systems would enable real-time monitoring, yield prediction, and quality control, further enhancing the automation of fruit classification in agriculture. By building on these considerations to believe that the proposed model, leveraging DenseNet121 with SE and YOLOv8n, has the potential to make significant strides in the automation of fruit classification and quality control, offering a practical and efficient solution for real-world agricultural applications.

## 7 Conclusion and future work

This study introduced an advanced approach to date fruit classification by integrating DenseNet121 with a Squeeze-and-Excitation (SE) attention block, enhancing feature representation and classification accuracy. The incorporation of data augmentation improved generalization and reduced overfitting, while Nadam optimization further refined model performance. Unlike traditional DenseNet architectures, the SE attention mechanism allowed the model to focus on critical image features, leading to superior classification results. Experimental evaluations demonstrated that DenseNet121+SE achieved 98.25% accuracy, 98.02% precision, 97.02% recall, and a 97.49% F1-score, while YOLOv8n achieved 96.04% accuracy, 99.76% precision, 99.7% recall, and a 99.73% F1-score, confirming the robustness of the approach compared to existing architectures. For future work to explore real-time deployment of the model for automated quality control in the food industry. Additionally, integrating multi-modal data, such as hyperspectral imaging or thermal sensing, could further enhance classification accuracy. Expanding the dataset to include more fruit varieties and different environmental conditions will also improve model generalizability, making it a more versatile solution for precision agriculture.

## Data Availability

The original contributions presented in the study are included in the article/supplementary material. Further inquiries can be directed to the corresponding authors.
